# 933 Analyzing the Long-Term Psychological Outcomes of 9/11 Survivors

**DOI:** 10.1093/jbcr/iraf019.464

**Published:** 2025-04-01

**Authors:** Anna Vaeth, Nancy Qin, Lucy Wei, Makayla Kochheiser, Grant Black, Nicholas Vernice, David Janhofer, Philip Chang, Palmer Bessey, David Otterburn

**Affiliations:** Weill Cornell Medicine; Weill Cornell Medicine; Weill Cornell Medicine; Weill Cornell Medicine; Weill Cornell Medicine; Weill Cornell Medicine; Weill Cornell Medicine; Weill Cornell Medicine; Weill Cornell Medicine; Weill Cornell Medicine

## Abstract

**Introduction:**

The September 11th attacks were a unique disaster with numerous patients and extensive injury burden. The aim of this study was to provide an update on the long-term psychological recovery of victims treated at a burn center following the September 11th attacks.

**Methods:**

A mixed methods approach was completed using a cross-sectional analysis survey to study health outcomes and a qualitative interview for each patient. All patients were treated at our institution’s burn center for burn injuries sustained during the September 11th attacks. Interviews were reviewed for trends in psychological impacts.

**Results:**

Our study included four patients including three males and one female. The average age was 63 years (range:57-73) and average total body surface area burned was 33.1% (range:3%-80%). One patient was burned in the elevator in the North Tower and one patient was burned in the lobby of North Tower during the impact of American Airlines Flight 11. Two patients were burned outside by debris. When asked about emotional health, two patients reported feeling emotionally healthy at least most of the time while the other two patients reported feeling emotionally healthy some of the time. One patient reported trouble sleeping following the attacks. Two patients reported general anxiety, two patients reported flashbacks that have persisted, and two patients reported feeling survivor’s guilt. Three of the four patients felt comfortable on an airplane all the time and comfortable around tall buildings. Three endorsed the annual commemoration of the attacks as a positive reflective experience, while one patient reported that they dread the day because it reminds them of how many lives were lost. Of note, all patients reported that the burn center gave them the necessary counseling services and support to circumvent additional hardship during their recovery journey.

**Conclusions:**

Our study showed that burn victims from the September 11th attacks suffered a wide range of long-term psychological impacts including general anxiety, sleeping, flashbacks, and survivor’s guilt. Despite these challenges, most patients reported feeling emotionally healthy and comfortable around tall buildings and in airplanes.

**Applicability of Research to Practice:**

This study seeks to provide new information on how the intersection of personal injury and large-scale disaster affects long-term emotional recovery. The interviews allow us to gain a deeper understanding of each patient’s experience in the long-term recovery phases. Although the September 11th attacks was a unique disaster, this study helps us predict how patients may recover from similar large-scale disasters in the future.

**Funding for the Study:**

N/A

